# A Rare Occurrence of Spontaneous Closure of a Sigmoid Loop Colostomy and an Inevitable Ventral Hernia

**DOI:** 10.7759/cureus.21161

**Published:** 2022-01-12

**Authors:** Ravikiran Thota, Murugappan Nachiappan, Srikanth Gadiyaram

**Affiliations:** 1 Surgical Gastroenterology & Minimally Invasive Surgery, Sahasra Hospital, Bengaluru, IND

**Keywords:** case report, symptomatic, sigmoid colostomy, hernia, loop stoma, spontaneous closure

## Abstract

An intestinal stoma is an opening of the intestinal tract onto the anterior abdominal wall. It is a commonly performed surgical procedure done for various benign and malignant pathologies. The construction of the stoma is temporary or permanent. Loop stoma is usually performed to divert the faecal stream for protection of the downstream anastomosis. They are usually reverted once the purpose of their creation is served. Spontaneous closure is a rare event that could result from a gradual stomal retraction. However, a normal bowel with no distal obstruction would be a prerequisite for it to be asymptomatic. Here, we report a case of spontaneous closure of a diversion loop sigmoid colostomy which had a delayed presentation. This is the second case of spontaneous closure of a sigmoid loop colostomy and the first report on the management of ventral hernias following spontaneously closed stoma in the English literature to the best of our knowledge.

## Introduction

Spontaneous closure of a stoma is a rare occurrence [1]. It is thought to result from a gradually progressive stomal retraction. Retraction of the stoma can be seen in the immediate post-operative period or several months after the procedure. Stomal retraction can occur in up to 40% of patients [2]. The factors predisposing to this in the early post-operative period include inadequate mobilization of the bowel during the creation of the stoma, thickened mesentery and edematous bowel when a stoma is fashioned for an emergency diversion in the presence of peritoneal inflammation, and malnutrition leading to mucocutaneous separation among others. Stomal retraction below the fascial level in the immediate post-operative period requires surgical intervention to prevent peritonitis. The increase in subcutaneous fat from weight gain can lead to stomal retraction later on [3]. Spontaneous closure of the stoma can result from gradual progress. However, a normal bowel with no distal obstruction is a prerequisite for it to be asymptomatic. Herein we report a case of spontaneous closure of a diversion loop sigmoid colostomy which had a delayed presentation. To the best of our knowledge, this is the second reported case of spontaneous closure of a sigmoid loop colostomy and the first report on the management of a ventral hernia following spontaneously closed stoma in the English literature.

## Case presentation

**Figure 1:**
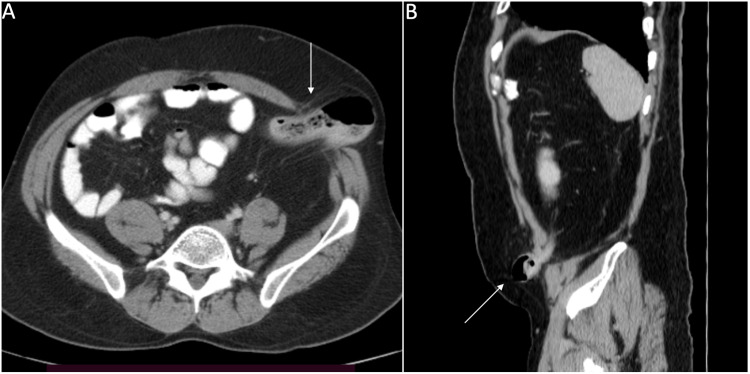
Contrast-enhanced CT images. A: Axial image showing the herniating sigmoid colon and the defect in the abdominal wall. B: Sagittal image showing the protruding sigmoid colon.

A 42-year-old male with a past medical history of Hirschsprung's disease presented to our outpatient department with worsening constipation over the last ten years. He was submitted to a Duhamel procedure with a diverting sigmoid loop colostomy at the age of 15 years. Following this, he had an unusual history of spontaneous closure of the colostomy at eight weeks post-surgery with normal passage of stool per rectum. After 15 years, he developed worsening constipation, which was managed with an increasing dose of laxatives. Evaluation with colonoscopy showed a sigmoid stricture and a pseudo-diverticulum formation proximal to the stricture. There was no gross mucosal abnormality at the site of narrowing and biopsy revealed chronic granulation tissue. The biopsies distal and proximal to this site showed ganglion cells in the submucosa. A contrast-enhanced CT of the abdomen revealed a 4cm x 3cm ventral hernia at the previous colostomy site, with the sigmoid colon reaching the level of the skin (Figure 1A, 1B). Systematic management was planned after a discussion with the patient. He was submitted to a laparoscopic segmental sigmoid colectomy with an anatomical repair of the muscle defect as the first step followed by a mesh hernioplasty.

**Figure 2:**
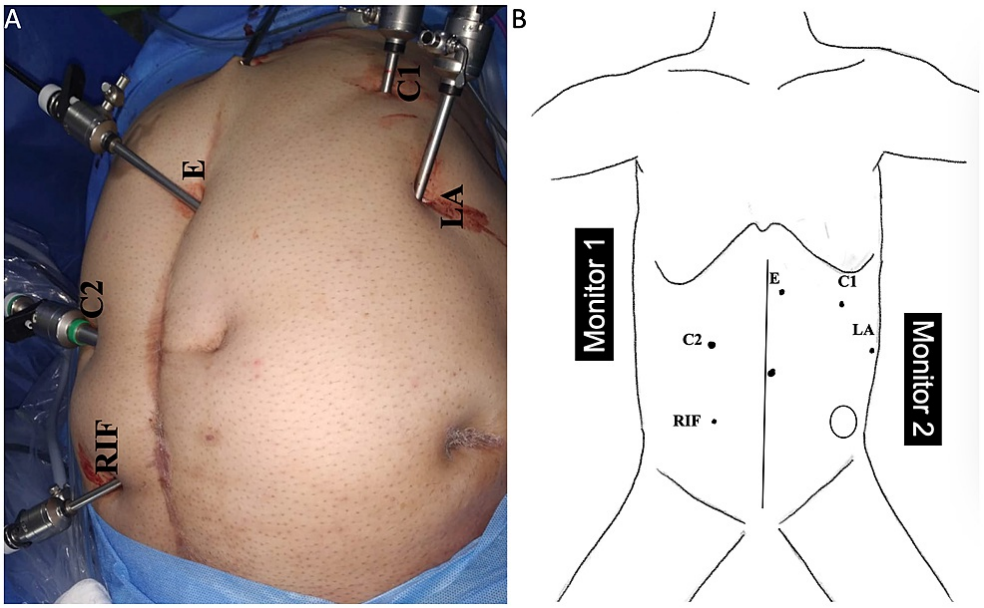
Port positions. A: Operative image showing the port sites. B: An illustration of the port sites. C1: 5mm Palmer’s point port was used for creating pneumoperitoneum and as a camera port during initial adhesiolysis, LA: 5mm port in left anterior axillary line was used for initial adhesiolysis in the epigastric region, E: 5mm port placed in the epigastrium was used as a left-hand working port during the latter part of the mobilization, C2: 10mm port placed in the mid-clavicular line to the right of the umbilicus was used as camera port during the latter part of colonic mobilization and stoma takedown, RIF: 5mm port used as a right-hand working port, Vertical line in the illustration: The previous midline surgical scar, Circular outline in the illustration: Scar of the stoma site

The procedure was completed laparoscopically. A closed pneumoperitoneum was established with Veress needle through Palmer’s point and a 5mm port was inserted. Another 5mm port was inserted in the left anterior axillary line. After adhesiolysis in the epigastric region, a third 5mm port was inserted in the epigastrium. Omental and small bowel adhesions to the parietal wall were lysed by sharp dissection with scissors. Subsequently, a 10mm port was inserted to the right of the umbilicus in the mid-clavicular line. A fifth port (5mm) placed in the right iliac fossa served as the right-hand working port for the surgeon (Figure 2A, 2B).

**Figure 3:**
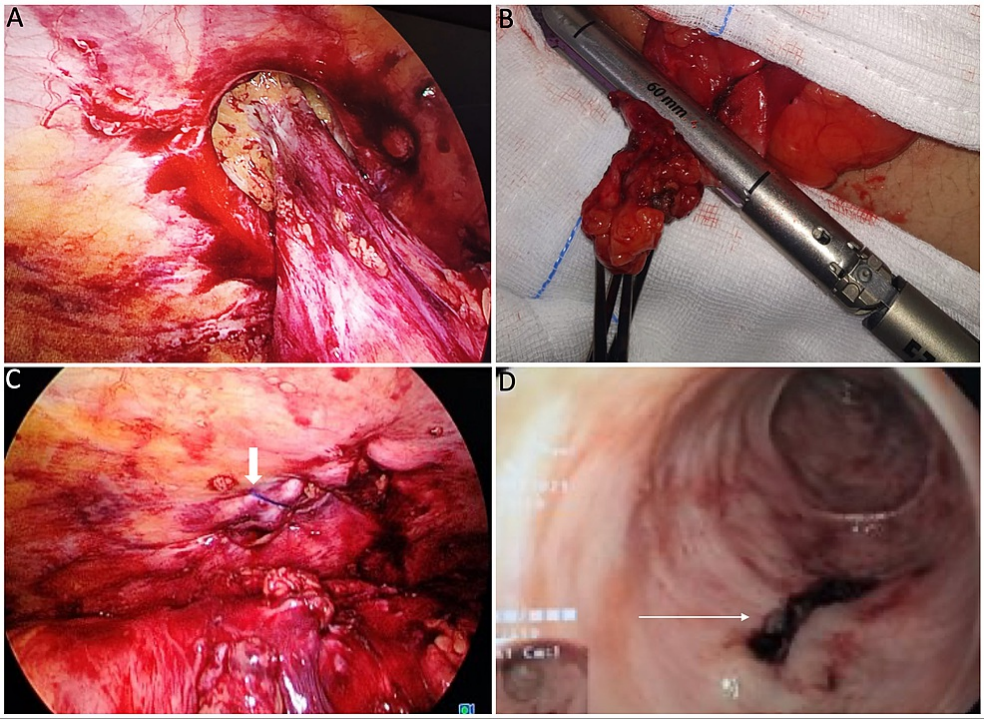
A: Defect in the abdominal wall at the previous stomal site with the adherent sigmoid colon after attempted dissection. B: Skin along with the part of the bowel wall staple excised with Endo GIA 60mm stapler medium/thick reload. C: Defect approximated with prolene (block arrow). D: Intra-operative colonoscopy showing the integrity of staple line (line arrow) and no compromise in the bowel lumen.

After the initial entry to the peritoneal cavity, adhesiolysis were performed with the surgeon on the left of the patient and the camera assistant to his left. However, the sigmoid colon proximal and distal to the herniated site was mobilized from the right side (Figure 3A). An elliptical incision was made over the previous colostomy site scar and the segment of the colon along with the adherent skin was delivered out. Careful inspection of this delivered colon showed a diverticular outpouching densely adherent to the scar of the previous colostomy. A staple resection of this diverticulum was done with an Endo GIA (Medtronic, Minneapolis, USA) 60mm medium/thick purple reload (Figure 3B). This did not result in a narrowing of the sigmoid colon at the stapled site. In view of a clean-contaminated wound, we performed an anatomical repair of the muscle defect with a plan of mesh hernioplasty during follow-up, if required. This was achieved by the Smead Jones suturing technique, which is our preferred method of stoma site closure. The skin was closed with interrupted non-absorbable sutures. A re-laparoscopy was done to provide a thorough lavage, confirm hemostasis, and check the wound closure (Figure 3C). An intra-operative colonoscopy showed no luminal compromise. It also confirmed the integrity of the staple line (Figure 3D).

The post-operative period was uneventful. The patient was started on an oral diet on the first post-operative day and discharged by the third post-operative day. On follow-up at six months, there was no evidence of a hernia.

## Discussion

Spontaneous closure of a loop colostomy is a rare occurrence, underlying mechanisms of which are poorly understood [1]. The reported studies cite retraction of the stoma site as a prelude to spontaneous closure. Pandit et al. have hypothesised that it is akin to the spontaneous closure of entero-cutaneous fistula [4]. The stoma, which is an iatrogenic end fistula, is converted to a lateral fistula gradually by stomal retraction, given that the distal bowel passage is intact and healthy. Akin to the closure of the lateral fistula, with time there can be spontaneous closure of the stomal site. The factors predisposing to stomal retraction in the early post-operative period include inadequate mobilization of the bowel during the creation of the stoma, thickened mesentery and edematous bowel when a stoma is fashioned for an emergency diversion in the presence of peritoneal inflammation, malnutrition leading to mucocutaneous separation, ischemic necrosis at the stoma site, and an excessive weight gain. Furthermore, the role of technical factors such as fascial fixation and the use of support rods is unclear. Our patient had the stoma created in an elective setting as a part of the primary surgery elsewhere. There is no suggestion of any stoma related problems in the immediate post-operative period. The patient had a significant increase in weight after the surgery, which if not in entirety, could have contributed to the stomal retraction and subsequent closure. To the best of our knowledge, this is the second case of spontaneous closure of a sigmoid loop colostomy to be reported in the English literature.

Spontaneous closure of a colostomy stoma is a serendipitous event for the patient in whom there is no pathology of the distal bowel, thus avoiding an elective surgery for stoma closure [5]. Further, the risk of incisional hernia at the stomal site is ever-present after a spontaneous closure given the fascial defect at the closure site. This may not be apparent immediately post-closure, however, and can manifest in a delayed fashion like in our patient.

The reported incidence of incisional hernia following stoma closure is around 30% [6]. This risk is even higher with spontaneous closure of stoma since the muscle defect persists. Also, with routine activities, straining, weight gain over a period, there can be a gradual widening muscular defect. The presence of a muscle defect greater than 2cm mandates a mesh hernioplasty in the vast majority of cases.

There are no reports in the literature on the management of ventral hernias following spontaneously closed stoma to the best of our knowledge. The concern specific to this problem is the bowel just below the skin, which can be breached during hernia repair. This would convert the wound into a clean-contaminated wound, increasing the risk of infection to a mesh used for hernia repair. This led us to offer repair as a two-stage procedure for our patient.

In our patient, there was a mechanical obstruction because of the kink of the bowel at the site at which it had adhered to the skin, seen as stricture with pseudo-diverticulum on colonoscopy. This was resected using a stapler. Although contamination would be limited by the use of a stapling device, the wound would still qualify as a clean-contaminated wound. Therefore, an anatomical repair was undertaken. The use of biological mesh to prevent an incisional hernia at a stoma closure site in a retro rectus position has been reported by Sepehr Lalezari et al., albeit in a few patients [7].

In summary, though a spontaneous closure of a loop colostomy is rare and may be seen as a fortuitous outcome in the short term, the inevitable consequence of a ventral hernia at the site would mandate an elective or emergency repair in the long term. Patients with a spontaneous closure should thus be counselled accordingly.

## Conclusions

Spontaneous closure of the stoma is a rare occurrence. The underlying mechanisms are poorly understood. Even though it can be a serendipitous event preventing a surgery in a few, a ventral hernia is inevitable. This can present in a delayed fashion with mechanical obstruction secondary to bowel adhesions. These patients need to be followed in the long-term for a ventral hernia at the site of previous stoma and are best served by an elective repair.
